# Synchronization of the Processes of Autonomic Control of Blood Circulation in Humans Is Different in the Awake State and in Sleep Stages

**DOI:** 10.3389/fnins.2021.791510

**Published:** 2022-01-12

**Authors:** Anatoly S. Karavaev, Viktoriia V. Skazkina, Ekaterina I. Borovkova, Mikhail D. Prokhorov, Aleksey N. Hramkov, Vladimir I. Ponomarenko, Anastasiya E. Runnova, Vladimir I. Gridnev, Anton R. Kiselev, Nikolay V. Kuznetsov, Leonid S. Chechurin, Thomas Penzel

**Affiliations:** ^1^Department of Basic Research in Neurocardiology, Institute of Cardiological Research, Saratov State Medical University, Saratov, Russia; ^2^Smart Sleep Laboratory, Saratov State University, Saratov, Russia; ^3^Laboratory of Nonlinear Dynamics Modeling, Saratov Branch of the Institute of Radio Engineering and Electronics of Russian Academy of Sciences, Saratov, Russia; ^4^LUT School of Engineering Science, LUT University, Lappeenranta, Finland; ^5^Coordinating Center for Fundamental Research, National Medical Research Center for Therapy and Preventive Medicine, Moscow, Russia; ^6^Faculty of Mathematics and Mechanics, St. Petersburg State University, St. Petersburg, Russia; ^7^Institute for Problems in Mechanical Engineering RAS, St. Petersburg, Russia; ^8^Interdisciplinary Sleep Medicine Center, Charité - Universitätsmedizin Berlin, Berlin, Germany

**Keywords:** autonomic control, cardiovascular system, sleep studies, synchronization, apnea, interbeat intervals, blood pressure

## Abstract

The influence of higher nervous activity on the processes of autonomic control of the cardiovascular system and baroreflex regulation is of considerable interest, both for understanding the fundamental laws of the functioning of the human body and for developing methods for diagnostics and treatment of pathologies. The complexity of the analyzed systems limits the possibilities of research in this area and requires the development of new tools. Earlier we propose a method for studying the collective dynamics of the processes of autonomic control of blood circulation in the awake state and in different stages of sleep. The method is based on estimating a quantitative measure representing the total percentage of phase synchronization between the low-frequency oscillations in heart rate and blood pressure. Analysis of electrocardiogram and invasive blood pressure signals in apnea patients in the awake state and in different sleep stages showed a high sensitivity of the proposed measure. It is shown that in slow-wave sleep the degree of synchronization of the studied rhythms is higher than in the awake state and lower than in sleep with rapid eye movement. The results reflect the modulation of the processes of autonomic control of blood circulation by higher nervous activity and can be used for the quantitative assessment of this modulation.

## Introduction

Arterial baroreflex plays an important role in the functioning of the autonomic nervous system (Subramanian et al., 2019). It is involved in the regulation of blood pressure (BP) (Osborn et al., 2005; Albaghdadi, 2007), heart rate (HR) (Lanfranchi and Somers, 2002), and respiration (Guyenet, 2014). According to a number of studies, the baroreflex and the processes of autonomic control determine the complex dynamics of blood circulation, in particular, the non-linear dynamics of heart rhythm (Tan, 2013; Ernst, 2017; Karavaev et al., 2019). It is known that autonomic control loops are sensitive to changes in the physiological state of healthy subjects (Ivanov et al., 1999; Valente et al., 2018; Ishbulatov et al., 2020). There is a significant change in baroreflex functions with aging (Sharma and Goodwin, 2006; Schumann et al., 2010; Shiogai et al., 2010; Elliott et al., 2016; Parashar et al., 2016; Natarajan et al., 2020; Pietri and Stefanadis, 2021). Impaired baroreflex regulation and impaired heart rate variability often accompany cardiovascular diseases (Kiselev et al., 2012a,b; Ponomarenko et al., 2013; Swenne, 2013; Karavaev et al., 2016). Therefore, the baroreflex sensitivity and the activity of autonomic control processes are often used to assess the physiological state of subjects and act as the markers of the development of pathologies of various systems of the body (Lázaro et al., 2019; Hadaya and Ardell, 2020; Wessel et al., 2020).

The regulation of the cardiovascular system and respiration is influenced by cortical structures that influence the baroreflex regulation under normal conditions (Furness, 2006; Duschek et al., 2013; Jerath and Beveridge, 2020) and during the development of pathologies (Lewis et al., 2006; Molkov et al., 2014). Considerable attention of researchers is paid to the peculiarities of baroreflex regulation and autonomic control of cardiovascular system during sleep (Brandenberger et al., 2003). The analysis of experimental signals in such studies involves the use of different methods for the assessment of baroreflex function and autonomic control (Shaffer and Ginsberg, 2017). Historically, the first are methods of statistical, correlation, and spectral analysis of RR-intervals (Task Force of the European Society of Cardiology [TFESC], 1996), which reflect the processes of frequency modulation of the HR by the autonomic control loops (Prokhorov et al., 2021). The analysis of RR-intervals traditionally includes the study of the so-called Low-Frequency (LF) band (0.04–0.14 Hz), which is mainly associated with the processes of sympathetic control of heart rate (Wagner and Persson, 1998), and the High-Frequency (HF) band (0.14–0.40 Hz), which is associated with the processes of parasympathetic control (Task Force of the European Society of Cardiology [TFESC], 1996; Lewis et al., 2006). Information about sympathetic regulation of blood pressure can be obtained by analyzing blood pressure signals in the LF-band (O’Leary and Woodbury, 1996; Elstad et al., 2011; Orini et al., 2012). At the same time, the frequency components of the blood pressure signal in the HF-band are mainly associated with the mechanical conduct of the respiration process into the vessels (Bernardi et al., 1996; Dash et al., 2010; Javed et al., 2010).

Despite considerable interest in the problem, the existing number of works describing the activity of autonomic control loops in the awake state and in different sleep stages: rapid eye movement (REM) and non-REM (NREM) demonstrates some contradictory results. In particular, in a seminal study (Somers et al., 1993) it is noted that the activity of the sympathetic branch of the autonomic control system increases in the REM sleep and decreases in the stages of NREM sleep relative to the awake state. Other studies show a decrease (Berlad et al., 1993; Parmeggiani, 1994), increase (Hornyak et al., 1991; Somers et al., 1993; Van de Borne et al., 1994) or the absence of changes (Vanoli et al., 1995) in the activity of sympathetic regulation of blood circulation in REM sleep compared with NREM sleep (Elsenbruch et al., 1999) showed a statistical difference between spectral assessments of the signals of systolic blood pressure (SBP), diastolic blood pressure and RR-intervals in the awake state, NREM and REM sleep; and (Legramante et al., 2003) shows their significant fluctuation depending on the sequence of the REM and NREM stages during the night.

The study (Zemaityte et al., 1984) revealed increases in the activity of the parasympathetic branch of the autonomic nervous system during REM sleep and decreases in NREM sleep state relative to the waking state. Such observations are explained by the modulation of the activity of the centers of autonomic control by the processes of higher nervous activity (Van Roon et al., 2004; Cheng et al., 2010).

In addition to the use of typical spectral assessments, baroreflex sensitivity assessments are often used (Laude et al., 2004; La Rovere et al., 2008; Maestri et al., 2009). However, the effectiveness of its use for the diagnosis of different sleep stages remains an open question. Thus, an increase in the sensitivity of the arterial baroreflex in sleep was noted in Smyth et al. (1969), Conway et al. (1983), Pagani et al. (1988), Parati et al. (1988), and Van de Borne et al. (1994). Moreover, special experiments have shown that an increase in baroreflex sensitivity is not a simple consequence of decreasing in mean arterial pressure during sleep (Smyth et al., 1969). The authors of Bristow et al. (1969) and Nakazato et al. (1998) show no significant changes in different sleep stages.

Furthermore, it is worth noting, that although several methods have been developed to study baroreflex sensitivity in humans, most of these techniques are of limited value for daily practice and require specialized pharmacological or mechanical manipulations (Smyth et al., 1969; Eckberg et al., 1975; Palmero et al., 1981; Airaksinen et al., 1993; Bernardi et al., 1995; Mortara et al., 1997; Pinna et al., 2000).

Nevertheless, the study of baroreflex function and autonomic control during sleep is of great importance in patients and healthy subjects, and research interest in this issue does not decrease (Guilleminault et al., 1981; Lombardi et al., 2019).

Thus, although the analysis of heart rate variability properties seems to correlate with the psychophysical state of the patient and the baroreflex function is associated with sleep stages, the sensitivity of linear analysis methods is limited (Billman, 2013).

Therefore, more sensitive non-linear methods of analysis are used in recent years. Such methods aim at assessing different measures of complexity in RR-intervals and blood pressure signals (Pichot et al., 2016; Ishaque et al., 2021). Earlier in the work (Karavaev et al., 2009) we proposed a quantitative measure representing the total percentage *S* of phase synchronization between the LF oscillations in RR-intervals and BP (Karavaev et al., 2021). In the works (Kiselev et al., 2012a,b, 2016b), we showed that the previously proposed method (Karavaev et al., 2009) is promising for solving problems of medical diagnostics. This measure allows us to predict the development of complications of myocardial infarction (Kiselev et al., 2012a), to select the drug therapy for arterial hypertension (Kiselev et al., 2012b), and to obtain important information about the structure of interactions between the elements of autonomic control of the cardiovascular system (Kiselev et al., 2020). In work (Ponomarenko et al., 2021), we showed a change in the coherence of respiration processes and parasympathetic regulation of the heart rate in the HF-band in the awake state and different stages of sleep. However, studies of the synchronization of the processes of autonomic control of blood circulation in the LF-band with changes in the physiological state of the subjects during sleep and awake state remain unknown.

Therefore, the purpose of this work is to identify changes in the synchronization of processes of autonomic control of the HR and BP when analyzing the LF-components of RR-intervals and blood pressure signals in the awake state, REM and NREM stages of sleep, as well as to study the possibility of classifying such states during the analysis of synchronization and other assessments characterizing the properties of heart rate variability (Task Force of the European Society of Cardiology [TFESC], 1996) and baroreflex sensitivity (Pagani et al., 1988).

## Materials and Methods

### Object of Study

**FIGURE 1:**
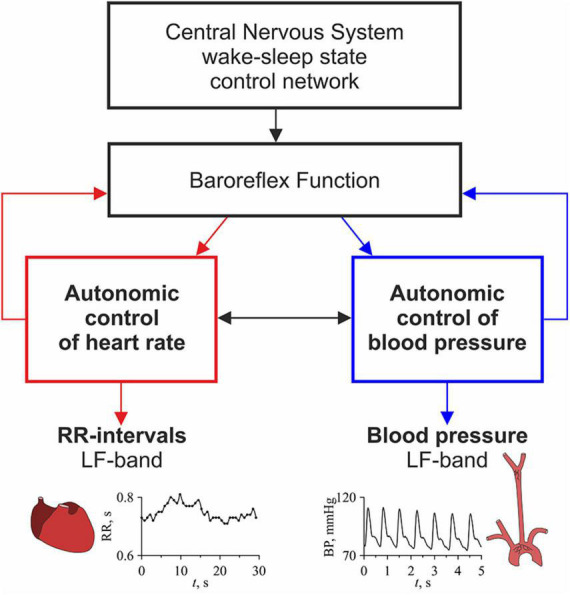
A simplified schematic illustration of the study.

Figure 1 shows a simplified schematic illustration of the object of study. The autonomic nervous system is actively involved in the control of HR and BP, using baroreflex functions (Task Force of the European Society of Cardiology [TFESC], 1996; Ringwood and Malpas, 2001). The work (Kiselev et al., 2020) shows, this control is provided by relatively independent processes that interact with each other. Information about the processes of autonomic control of HR and BP can be extracted from the LF-components of RR-intervals and BP signals (Task Force of the European Society of Cardiology [TFESC], 1996; Ringwood and Malpas, 2001; Prokhorov et al., 2003). It shows that these LF-components can be synchronized, and the degree of their synchronization correlates with the state of the cardiovascular system (Karavaev et al., 2009). It is known that the wake-sleep state control network of the central nervous system modulates the activity of the elements of the autonomic nervous system, and this influence changes during the transition from the awake state to sleep stages (Van Roon et al., 2004; Cheng et al., 2010). Thus, the degree of synchronization between the processes of autonomic control of HR and BP should reflect the effect of the wake-sleep state control network of the central nervous system on the autonomic nervous system.

### Experimental Data

We analyzed the electrocardiogram (ECG), BP, and respiration signals of 22 male patients (mean age 52 ± 10 years, body mass index 32.4 ± 6.0 kg/m^2^) obtained in a clinical study (Peter et al., 1989; Grote et al., 1995). All patients were hospitalized due to obstructive breathing disorders during sleep (mean respiratory disturbance index 47.2 ± 27.3 n/h), and arterial hypertension was also revealed in the patients. Recordings were made before the therapy.

The ECG signal was recorded in standard lead I according (Kligfield et al., 2007). The BP signal (time dependence of blood pressure during invasive arterial pressure monitoring) was recorded with an invasive monitoring system from the radial is artery. The respiration signal was recorded with a flowing oronasal airflow. All signals were filtered with the bandpass of 0.03–45 Hz and sampled at 100 Hz. For each subject, we extracted three 20-min fragments from the night record, one corresponding to the awake state, one to the stage S3 of NREM sleep, and one to REM sleep. The awake state and sleep stages were scored in accordance with the recommendations of Rechtschaffen and Kales (1968) by means of electro-oculogram, electroencephalogram, and electromyogram. The analyzed fragments did not contain artifacts or long periods of apnea.

### Total Percentage of Phase Synchronization

To analyze the degree of synchronization between the LF oscillations in HR and BP, we use a quantitative measure representing the total percentage *S* of phase synchronization of the studied processes. The method is based on the approach for detecting synchronization of complex systems from the analysis of their non-stationary time series (Karavaev et al., 2009; Kiselev et al., 2016a). Comparison of the proposed method with other known methods for detecting synchronization showed its higher sensitivity (Borovkova et al., 2020, 2021).

**FIGURE 2:**
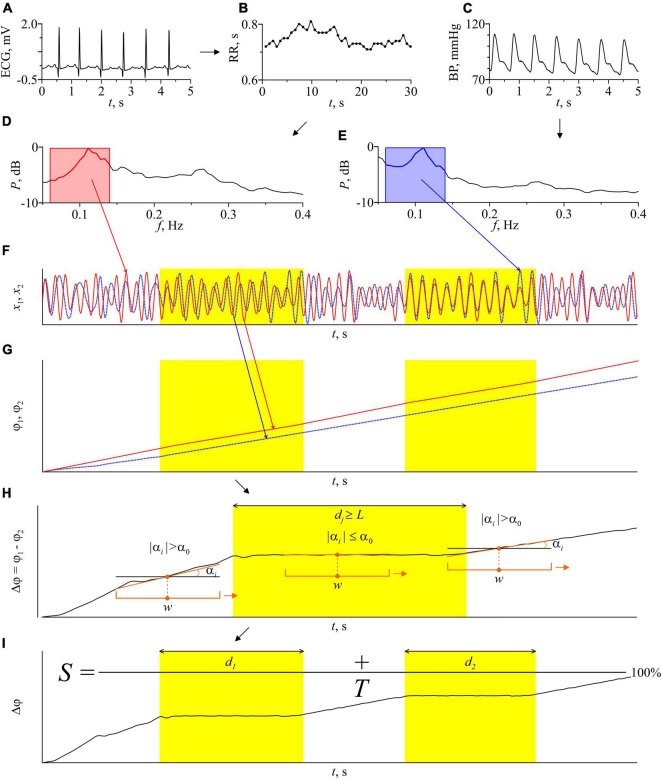
Schematic illustration of the method for estimating the total percentage *S* of phase synchronization. **(A)** ECG signal; **(B)** RR-intervals. Dots show experimental values of RR-intervals, interpolation by cubic β-splines is shown by a line; **(C)** the BP signal; **(D)** the power spectrum of the RR-interval signal; **(E)** power spectrum of the blood pressure signal. The bandwidth of the bandpass filters is marked in pink and blue on the power spectra graphs; **(F)** signals of RR-intervals and blood pressure filtered in the LF-band and normalized to unity amplitude; **(G)** instantaneous phases of the filtered signals of RR-intervals and blood pressure; **(H)** the instantaneous phases difference of the signals and an illustration of the automatic procedure for finding the epochs of synchronization. Orange bracket marks a window sliding along the time series Δϕ(*t*) with a shift of 1 discrete sample. *w* is the sliding window width. The orange line is a linear approximation of the time series Δϕ(*t*) in every sliding window. α_*i*_ is the slope of the approximating line in *i*th sliding window. α_0_ is the method parameter, maximum slope angle, *d*_*j*_ is the length of *j*th epoch of synchronization. Parameter *L* has the meaning of the minimum length of the synchronization epoch; **(I)** illustration of the procedure for calculating the index of the total percentage of phase synchronization *S*. *T* is the time series length, *d*_1_ and *d*_2_ are the length of the identified epochs of synchronization. The epochs of synchronization are shown in yellow.

Figure 2 illustrates the main stages of the method. From the experimental ECG signal we extract a sequence of RR-intervals, i.e., a series of time intervals between the two successive R peaks. To obtain equidistant time series from not equidistant sequence of RR-intervals, we approximate it with cubic *b*-splines and resampled with a frequency of 5 Hz. Then, we filter the sequence of RR-intervals and the signal of BP using a filter with the bandpass of 0.06–0.14 Hz. After that, we resample the filtered BP signal with a sampling frequency of 5 Hz. The filtered signals are normalized so that their maximum amplitude is equal to unity.

Next, we define the instantaneous phases ϕ_1_(*t*) and ϕ_2_(*t*) of the filtered RR-intervals and BP signal, respectively, as the angle of rotation of the radius vector on the plane $\left(x\,\left(t\right),\tilde{x}\,\left(t\right)\right)$, where *x*(*t*) is the analyzed signal and $\tilde{x}\,\left(t\right)$ is the Hilbert transform of *x*(*t*),


(1) $$\tilde{x}\,\left(t\right)=\frac{1}{\pi }\,P.V.\int_{-\infty }^{+\infty }\frac{x\,\left(\tau \right)}{t-\tau }\,d\tau ,$$

where P.V. means that the integral is taken in the sense of the Cauchy principal value on a time series (Rosenblum and Kurths, 1998). Then, we calculate the difference of instantaneous phases ϕ_1_(*t*) and ϕ_2_(*t*):


(2) $$\Delta \,\phi \,\left(t\right)=\phi_{2}\,\left(t\right)-\phi_{1}\,\left(t\right).$$

The presence of 1:1 phase synchronization of two processes is defined by the condition:


(3) $$\left|\phi_{2}\,\left(t\right)-\phi_{1}\,\left(t\right)\right|<C,$$

where *C* is a constant (Pikovsky et al., 2001). Thus, in the epochs of synchronization, Δϕ(*t*) demonstrates horizontal plateaus, the oscillations on which are determined only by the measurement noise.

For automatic detection of phase synchronization epochs, we use the following algorithm. A window with a width *w* moves along the time series of Δϕ(*t*) with a shift of one sample (0.2 s). Using the method of least squares, we linearly approximate Δϕ(*t*) in each *i*th moving window by calculating a slope α_*i*_. The presence of synchronization is defined by the condition |α_*i*_|≤α_0_, where α_0_ is the method parameter. To reduce the influence of random fluctuations of instantaneous phases, we use an additional condition *d*_*j*_≥*L* for the detection of phase synchronization, where *d*_*j*_ is the length of the *j*th epoch of synchronization and *L* is the method parameter. Specialized statistical tests carried out in the study (Borovkova et al., 2020, 2021) allow us to choose the following parameters of the method: *w* = 22 s, α_0_ 0.004 rad, *L* = 10 s. These values are used throughout the paper.

A sufficiently wide *w* window (more than 2 characteristic periods of oscillations) allows one to reduce the influence of phase noise. The method’s algorithm assumes that the epoch is identified as an epoch of synchronization if the slope of the approximating straight line remains below the threshold value for a continuous-time interval with a length of at least *L* (about 1 characteristic period). In this case, the window *w* is shifted each time by one sample of discrete-time. Such conditions make it possible to increase the accuracy of diagnostics of the boundaries of the epoch of synchronization when moving the window *w* between synchronous and asynchronous epochs. Then we calculate the total length of the identified epochs of phase synchronization and express it as a percentage relative to the time series length of the entire record. The total percentage of phase synchronization is calculated as follows:


(4) $$S=\frac{\sum_{j=1}^{N}d_{j}}{T}\times 100\%,$$

where *T* is the time series length of the entire record and *N* is the number of epochs of synchronization. The calculation of the phase synchronization index *S* uses the LF-filtered time series of RR-intervals and BP as a whole: 20-min time series (6000 samples at a sampling rate of 5 Hz).

The method for calculating measure *S* is implemented using the Python programming language. The program is available for free use at^1^.

### Statistical Significance of the Index *S*

Signals of complex systems of biological nature are usually non-linear, non-stationary, and susceptible to noise and artifacts of different nature. Therefore, each values of index *S* obtained from experimental data was accompanied by the estimate the statistical significance. For each 20-min pair of ECG and BP signals, we generate a statistical ensemble of 100 pairs of surrogate time series by randomizing the phases of the Fourier-harmonics of analyzed signals in accordance with the method proposed in Theiler et al. (1992) and Schreiber and Schmitz (1996). This method for surrogate data preparation preserves periodogram of the experimental signals, but destroys couplings between them. Then, we calculate a total percentage of phase synchronization *S*_*k*_, *k* = 1,…,100 for each *k*th pair of surrogates and sorted *S*_*k*_ values in ascending order. The measure *S* calculated from the experimental signals is considered statistically significant (*p* < 0.05) if it is greater than the 95th *S*_*k*_ value calculated from surrogate data.

### Heart Rate Variability Indices

Some well-known works (Smyth et al., 1969; Pagani et al., 1988; Vanoli et al., 1995; Nakazato et al., 1998; Elsenbruch et al., 1999; Legramante et al., 2003) show a change in the baroreflex function and, as a consequence, the values of common assessments characterizing the properties of heart rate and blood pressure change during the transition from the awake state to different stages of sleep. Linear methods of spectral and statistical analysis of signals are often used for these investigations (Task Force of the European Society of Cardiology [TFESC], 1996). The following assessments are widely used to separate the awake state and different stages of sleep: *X*_*mean*_ (ms) - mean length of RR-intervals, *SDNN* - the standard deviation of length of the RR-intervals, *HF*_*RR*_ (ms^2^) - the power of the spectrum of the HF components of the RR-intervals and BP, *LF*_*RR*_ (ms^2^) - the power of the spectrum of the LF components of the RR-intervals. We calculate these assessments in this work. We used the first 5 min of the RR-interval signal. The calculation of the assessments of heart rate variability was carried out by the well-known methodological recommendations (Task Force of the European Society of Cardiology [TFESC], 1996).

### Baroreflex Sensitivity Index

Baroreflex sensitivity (BRS) is also a recognized tool for assessing the autonomic control of the cardiovascular system.

In this work, the assessment of the baroreflex sensitivity was carried out based on spectral analysis of the signal of RR-intervals and systolic blood pressure and was used along with other typical tools for diagnosing the stages of sleep and the awake state (Smyth et al., 1969; Pagani et al., 1988; Legramante et al., 2003). BRS assessments are often based on calculating the power spectral density of the RR-intervals and SBP signals in the LF and/or HF frequency bands (Pagani et al., 1988; Legramante et al., 2003; Dietrich et al., 2010). In Dietrich et al. (2010), results showed similar values of BRS assessments carried out by different methods. Therefore in this paper, BRS was assessed by calculating the alpha index in accordance with the recommendations (Pagani et al., 1988).

In this work the BRS was assessed based on the signals of the RR-intervals and the signal of SBP. To calculate the assessment of BRS, we isolated a non-equidistant sequence of peaks from invasive BP signal (one peak per cardiocycle - at the moment of systole). Subsequently, this non-equidistant series of the SBP signal and the RR-intervals signal was interpolated with a cubic β-spline up to a sampling frequency of 5 Hz. The power spectra were calculated from them, and the integrated power in the LF-band (0.04–0.15 Hz) was estimated.


(5) $$B\,R\,S=\sqrt{\int_{f_{1}}^{f_{2}}S\,\left(f_{L\,F_{R\,R}}\right)\,df/\int_{f_{1}}^{f_{2}}S\,\left(f_{L\,F_{S\,B\,P}}\right)\,df},$$

where *f*_1_ 0.04 Hz, *f*_2_ 0.15 Hz, is $\int_{f_{1}}^{f_{2}}S\,\left(f_{L\,F_{R\,R}}\right)\,df$ the integrated power of the spectrum of the LF-component of the RR-intervals, $\int_{f_{1}}^{f_{2}}S\,\left(f_{L\,F_{S\,B\,P}}\right)\,df$ is the integrated power of the spectrum of the LF-component of the SBP.

This assessment of *BRS* estimate is based on the assumption of a high degree of linear correlation between RR-intervals and SBP variability in the LF-band. This method is simple enough to quickly estimate BRS based on standard spectral characteristics.

### Statistical Analysis

We used receiver operating characteristic (ROC) to assess the quality of the binary classification of calculated indices for different pairs of states (REM vs awake, REM vs. NREM, and NREM vs. awake).

Let us illustrate the procedure for constructing ROC-curves by analyzing the *S* index as an example. To construct the ROC curve for each pair, we went through the threshold value of the parameter of total percentage of phase synchronization *S*_0_ from 0 to 100% with step 1%. Each threshold value *S*_0_ we compared with grades *S*_*i*_, *i* = 1,..,22 for all subjects in the first (in each matched pair) state and *S*_*j*_, *j* = 1,..,22 – in the second state. To estimate TPR, we calculated the split of results *S*_*i*_> *S*_0_. To estimate FPR we calculated the split of results *S_*j*_* > *S*_0_. Each point on the ROC curve shows the relationship between TPR and FPR for each threshold value *S*_0_. Comparison of the ROC curves was carried out in the course of calculating the characteristic - Area Under the Curve (AUC).

Similarly, we constructed ROC curves for the other comparable assessments. At the same time, for the *X*_*mean*_ and *HF*_*RR*_ indices, for classifying states in accordance with the results *a priori* known from Oh et al. (2019), it was assumed that during the transition from the awake and REM sleep to the deep sleep, the assessments should increase, and for other cases-decrease.

For each ROC-curve, we found the threshold value of the index corresponding to the put off point (the point closest to the point TPR = 1.0, FPR = 0.0). For put off points, we calculated the odds ratio and *p*-value.

To check the reliability of differences in the estimates of the mean values of assessments for different stages, we used the Kruskal-Wallis test (Kruskal, 1952), which takes into account multiple testing, and the Mann-Whitney *U*-test (Mann and Whitney, 1947), which does not take into account the correction for multiple testing but allowing for pairwise comparison.

## Results

**FIGURE 3:**
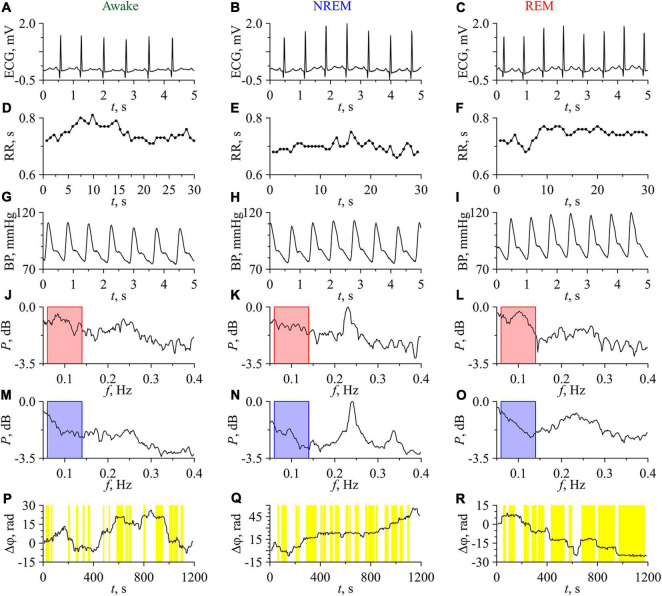
Experimental signals, their Fourier power spectra and the difference of instantaneous phases for the subject No. 1 (No. C04Z1N2) in the awake state (first column), NREM sleep (second column), and REM sleep (third column). **(A–C)** ECG signals; **(D–F)** RR-intervals. Experimental values are shown with dots and interpolation by cubic β-splines is shown with line; **(G–I)** BP signals; **(J–L)** Fourier power spectra of RR-intervals; **(M–O)** Fourier power spectra of BP signals. The bandpass of filtering is shown in the power spectra plots in pink and blue; **(P–R)** Phase differences. Epochs of phase synchronization are shown in yellow.

Figure 3 shows the experimental signals and the results of their analysis for one of the subjects in the awake state, NREM sleep, and REM sleep. Figures 3A–C present the ECG signals in the awake state, NREM sleep, and REM sleep, respectively.

RR-intervals show low variability, that typical in subjects at rest (Figures 3D–F). BP signals in the awake state, NREM sleep, and REM sleep are presented in Figures 3G–I, respectively. The values of BP correspond to those observed in healthy subjects (Young et al., 2020). The power spectra of the RR-intervals are shown in Figures 3J–L, while the power spectra of the BP signals are shown in Figures 3M–O. These power spectra are normalized to the maximum value in the three considered states, which takes place for NREM sleep. The characteristic peaks in the LF-band of power spectra are associated with the processes of autonomic control and baroreflex activity, while the peaks in the HF-band are associated with the respiration.

In the spectra of RR-intervals, the power in the LF-band in the awake state is higher than in NREM sleep and lower than in REM sleep. This is consistent with the known results (Somers et al., 1993; Kantelhardt et al., 2002). On the contrary, the power in the HF-band in the awake state is lower than in NREM sleep and higher than in REM sleep. This result agrees well with other studies (Zemaityte et al., 1984; Bartsch et al., 2012).

In the power spectra of BP signals, the HF-component is most pronounced in NREM sleep. Oscillatory activity in the HF-band of BP signal is usually related to the respiratory driven BP oscillations through changes in the intra-thoracic pressure if the respiratory frequency remains within the normal limits (Dornhorst et al., 1954; De Boer et al., 1987; Sleight et al., 1995; Conci et al., 2001). The study of such effects was beyond the scope of this paper.

Figures 3P–R depict the dependences Δϕ(*t*) for the awake state, NREM sleep, and REM sleep, respectively. The length of phase synchronization epochs in REM sleep is noticeably greater than in other two states. In the awake state, Δϕ(*t*) slowly fluctuates around 0. In NREM sleep, a slow positive trend of Δϕ(*t*) is observed, which indicates that the instantaneous phase of LF-component in RR-intervals grows faster than the phase of LF-component in BP signal. In REM sleep, a negative trend of Δϕ(*t*) takes place that indicates the faster increase of the phase of BP signal LF-component with respect to the phase of LF-component in RR-intervals. However, the sign of trend in Δϕ(*t*) differs in different subjects. In Figure 3, the total percentage *S* of phase synchronization is 29.6% in the awake state, 43.3% in NREM sleep, and 65.8% in REM sleep. All these values are statistically significant.

**FIGURE 4:**
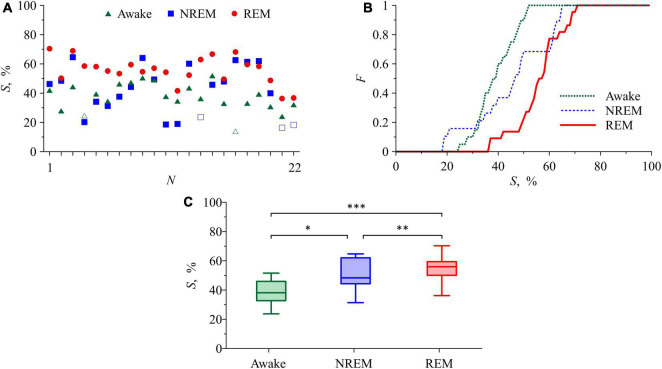
**(A)** *S* values in the awake state, NREM sleep, and REM sleep for each of the subjects. Statistically insignificant *S* values are shown with white squares and white triangles. **(B)** Distribution function for *S*. **(C)** Box-and-whisker diagrams for *S* values in the awake state, NREM sleep, and REM sleep. The box boundaries are the first and third quartiles, the horizontal line is the median, the whiskers are the minimum and maximum values. Figures **(B,C)** include only statistically significant *S* values (*p* < 0.05). The asterisks in panel **(C)** correspond to the *p*-level of intergroup differences, assessed using the Mann-Whitney *U*-test: *p*= 0.01 for “*” and “^**^” and *p*< 0.01 for “^***^”.

Figure 4 presents the results of the analysis of synchronization between the studied processes of autonomic control of blood circulation. In Figure 4A, the *S* values in the awake state, NREM sleep, and REM sleep are shown for each of 22 subjects. In all subjects, the *S* values in REM sleep are higher than in the awake state. In 18 subjects, the *S* values in REM sleep are higher than in NREM sleep. The difference between the *S* values in the awake state and in NREM sleep is less pronounced. From all *S* values in Figure 4A, only five values (two values in the awake state and three values in NREM sleep) are not significant. Figure 4B shows the distribution function *F* calculated for significant *S* values. In Figure 4C, box-and-whisker diagrams for *S* values are presented. From these figures it follows that, on average, *S* takes the highest values in REM sleep and the lowest values in the awake state. The most pronounced difference is observed between *S* values in the awake state and in REM sleep stage. In NREM sleep, the *S* values have high variance and are on average higher than in the awake state and lower than in REM sleep, Figure 4C.

The quantitative results of comparing the changes in the total percentage of phase synchronization *S* with known assessments in the awake state and different stages of sleep are presented in Table 1.

**TABLE 1:** The values of the calculated assessments in the awake state and in sleep stages.

Subject’s state	Awake	NREM	REM
*S*, %	38.46 (32.99; 45.31)	48.24 (42.08; 61.69)	56.1 (50.23; 59.58)
*X*_*mean*_, ms	901.83 (800.13; 1051.96)	982.15 (877.66; 1075.63)	932.72 (797.13; 1093.44)
*SDNN*, ms	62.02 (38.02; 90.46)	35.93 (26.87; 49.84)	60.26 (40.13; 93.33)
*HF*_*RR*_, ms^2^	83.84 (42.05; 150.47)	209.37 (97.07; 539.01)	75.23 (38.61; 305.85)
*LF*_*RR*_, ms^2^	397.57 (248.53; 722.90)	199.18 (98.20; 373.10)	365.06 (206.91; 1192.66)
*BRS*	15.14 (13.32; 17.93)	13.27 (11.35; 21.56)	15.98 (10.19; 19.80)

*Values are presented in the format: median (first quartile; third quartile).*

Table 1 shows that the index *S* demonstrates the best divergence of the distributions of values between awake state and REM sleep compared to other indices.

Table 2 gives the results of a pairwise test of the statistical significance of intergroup differences using the Mann-Whitney *U*-test and the Kruskal-Wallis test, which takes into account multiple testing.

**TABLE 2:** Statistical significance of intergroup differences in the awake state and in sleep stages using different assessments.

Indexes	*p*-values Mann–Whitney *U*-test			Kruskal-Wallis test
	REM vs. awake	REM vs. NREM	NREM vs. awake	
*S*, %	**<0.01**	**0.01**	**0.01**	**<0.01**
*X*_*mean*_, ms	0.49	0.65	0.18	0.42
*SDNN*, ms	0.63	**<0.01**	**0.01**	**<0.01**
*HF*_*RR*_, ms^2^	0.97	0.05	**0.01**	**0.03**
*LF*_*RR*_, ms^2^	0.99	**0.04**	**0.03**	0.05
*BRS*	0.95	0.70	0.82	0.96

*Pairwise comparisons were made using the Mann-Whitney U-test, multiple testing using the Kruskal-Wallis test. Statistically significant (p < 0.05) values are highlighted in bold.*

Tables 1, 2 show that pairwise testing with the Mann–Whitney *U*-test shows that the index *S* separates REM sleep and awake state, as well as REM sleep and NREM sleep. *SDNN* and *LF*_*RR*_ separate groups REM sleep and NREM sleep, as well as NREM sleep and the awake state. *HF*_*RR*_ separates group NREM sleep and the awake state. This is confirmed by the results of multiple testing using the Kruskal-Wallis test (Table 2), which confirms significant group differences for all of the listed indices, except for the *LF*_*RR*_.

Thus, only 4 out of 6 assessments demonstrated significant intergroup differences between the compared states for the experimental sample used. At the same time, only the *S* index made it possible to separate the REM sleep and the awake state groups significantly.

ROC analysis was carried out to assess the capabilities of the compared assessments when solving the problem of classifying of the awake state and sleep stages.

**FIGURE 5:**
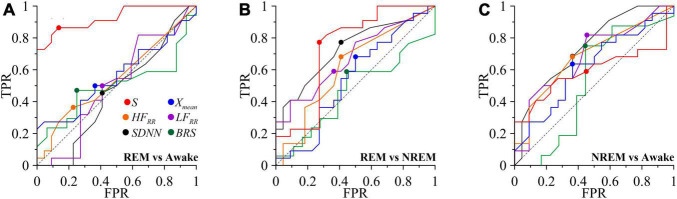
ROC curves for classifying the awake state and sleep stages. TPR is true positive rate and FPR is false positive rate. **(A)** REM and awake state. **(B)** REM and NREM. **(C)** NREM and awake state. The diagonal line is shown with black dashed line. Put off point is marked with colored dots.

Figure 5 shows the ROC-curves for the *S* index. The ROC analysis results for the index *S* are consistent with the results of the analysis of intergroup differences. *S* demonstrates a noticeably better ability among the compared indices to classify REM sleep and the awake state (red line in Figure 5A), slightly worse results for REM and NREM sleep (red line in Figure 5B), worst of all these assessments allows classifying NREM sleep and awake states (Figure 5C). Among other assessments, the *SDNN* and *HF*_*RR*_, *LF*_*RR*_ indexes can be noted, for classifying the REM sleep and NREM sleep (black, orange and purple lines, respectively, in Figure 5B) and NREM sleep and awake states (black, orange and purple lines, respectively, in Figure 5C). The *BRS* assessment shows the ability to classify NREM sleep and awake state (green line in Figure 5C).

The quantitative results of comparing the possibilities of classifying states using these assessments are shown in Table 3.

**TABLE 3:** Results of the ROC analysis for classifying the awake state and sleep stages for the compared assessments, as well as the *p*-value and odds ratio for the put off points of the ROC curves.

Indexes	Subject’s state	Point of ROC curve intersection with the diagonal					
		AUC	TPR	FPR	put off point	*p*-value	Odds ratio
*S*, %	REM vs awake	0.92	0.86	0.14	49	**<0.01**	40.1 (5.8–368.6)
	REM vs NREM	0.73	0.77	0.27	53	**<0.01**	9.1 (1.9–46.6)
	NREM vs awake	0.59	0.59	0.45	40	0.55	1.7 (0.4–6.8)
*Xmean*, ms	REM vs awake	0.56	0.50	0.36	904	0.54	1.8 (0.4–7)
	REM vs NREM	0.54	0.68	0.50	969	0.36	2.1 (0.5–8.8)
	NREM vs awake	0.62	0.64	0.36	929	0.13	3.1 (0.8–12.7)
*SDNN*, ms	REM vs awake	0.54	0.45	0.41	61	1.00	1.2 (0.3–4.7)
	REM vs NREM	0.74	0.77	0.41	663	**0.03**	4.9 (1.1–22.9)
	NREM vs awake	0.74	0.68	0.36	663	0.07	3.8 (0.9–16.1)
*HF*_*RR*_, ms^2^	REM vs awake	0.55	0.36	0.23	9903	0.51	1.9 (0.4–9)
	REM vs NREM	0.62	0.68	0.41	203	0.13	3 (0.8–13)
	NREM vs awake	0.69	0.68	0.36	103	0.07	3.8 (0.9–16.1)
*LF*_*RR*_, ms^2^	REM vs awake	0.50	0.50	0.41	353	0.77	1.4 (0.4–5.7)
	REM vs NREM	0.68	0.59	0.36	263	0.23	2.5 (0.6–10.3)
	NREM vs awake	0.69	0.82	0.45	293	**0.03**	5.4 (1.2–27.2)
*BRS*	REM vs awake	0.50	0.47	0.25	15.88	0.34	2.7 (0.5–15.4)
	REM vs NREM	0.47	0.59	0.44	14.36	0.61	1.8 (0.4–8.5)
	NREM vs awake	0.53	0.75	0.44	14.74	0.09	4.5 (0.8–26.7)

*Statistically significant (p < 0.05) values are highlighted in bold.*

Thus, Table 3 shows that the degree of synchronization of the processes of autonomic control of blood circulation with the calculation of the *S* index, in contrast to other assessments, makes it possible to classifying the state of REM sleep and the awake state. In Figure 5, the ROC-curve for this pair of states is higher than the other two ROC-curves. It gives good results TPR = 0.86 and specificity FPR = 0.14 for put off point (Table 3). The *S* and *SDNN* indexes differentiate between REM sleep and NREM sleep. The *SDNN* and *HF*_*RR*_ indices differentiate between REM sleep and NREM sleep. The *LF*_*RR*_ indice differentiate between NREM sleep and the awake state. Significant results (*p* < 0.05) are highlighted in bold.

## Discussion

The baroreflex function ensures the operation of the loops of autonomic control of blood circulation, which sensitively react to changes in the physiological state of the body. The studies (Mrowka et al., 2000, 2003; Shiogai et al., 2010; Bartsch et al., 2012) shows a statistically significant change in the cardiorespiratory synchronization and direction of coupling with aging. It is shown that the degree of cardiorespiratory synchronization in healthy subjects, on average, correlates well with the depth of sleep, being minimal in REM sleep and higher in deep sleep than in the awake state (Bartsch et al., 2012). Thus, the study of synchronization between the rhythms of the cardiovascular system gives important information about the state of the subjects and reflects physiological changes in the dynamics of the loops of baroreflex regulation in different stages of sleep.

In this paper, in different sleep stages, we study the phase synchronization of oscillations in the autonomic control loops of HR and BP, the activity of which is manifested mainly in the LF-band. The signals of these loops demonstrate complex chaotic non-stationary dynamics (Karavaev et al., 2019). To detect and quantify the synchronization between these signals, we used the previously proposed the method of synchronization analysis (Karavaev et al., 2009) and applied it to experimental signals of BP and RR-intervals. The method is implemented as freely distributed software.

The analysis of subjects in the awake state and different sleep stages allows us to reveal a number of interesting features. Despite the well-known effect of a decrease in the power of LF-oscillations in RR-intervals in NREM sleep (Somers et al., 1993), synchronization of the processes of autonomic control of blood circulation in NREM sleep increases with respect to the awake state (Figure 4). At the same time, we reveals the maximal synchronization during REM sleep, for which (Bartsch et al., 2012) reported the minimal values of cardiorespiratory synchronization. Our results confirm the conclusions obtained with the use of mathematical models in Van Roon et al. (2004) and Cheng et al. (2010) that the baroreflex function modulation by cortical structures has a complex independent nature. The change in the synchronization of the studied processes at different sleep stages can be explained by the change of the degree of effective interaction of these processes caused by such modulation. Presumably, the mechanisms of these interactions are realized through the brain stem structures (nucleus tract us solitarius, dorsal motor nucleus, nucleus ambiguous, and others), which interact with each other and with the structures of the cerebral cortex (Van Roon et al., 2004; Guyenet, 2014).

The potential of using the analysis of autonomic processes instead of the analysis of electroencephalograms is noted in Mayer et al. (2020) in the study of cardiorespiratory interaction. Currently, the classification of stages 1–3 of deep NREM sleep is successfully carried out by analyzing the instantaneous powers of various frequency components in electroencephalograms (Hassan and Subasi, 2017; Nakamura et al., 2017; Gupta and Pachori, 2021). At the same time, respiration, cardiac activity, and muscle tone also change significantly with the onset of NREM sleep (Flores et al., 2007; Noble and Hochman, 2019; Vanneau et al., 2021). However, the state of REM sleep is still reliably diagnosed only by assessing the eye dynamics and accompanying muscle activity (Liu et al., 2020). Some studies demonstrate certain changes in muscle activity and electrical activity of the brain, but these changes are not specific and very individual. In practice, the classification of REM sleep by automatic systems is very difficult (Cesari et al., 2018; Tripathy and Acharya, 2018). Nevertheless, it seems that this state may be described by analyzing the dynamics of various physiological signals, and not just based on the assessment of the appearance of eye movements.

The importance of this problem is emphasized by the fact that changes in the quality and quantity of REM sleep stages correlate with statistical assessments of mortality and the development of pathological conditions (Galbiati et al., 2019; Leary et al., 2020). At the same time, the known approaches based on estimates of linear spectral and statistical characteristics demonstrate a low sensitivity of the classification of REM sleep. The measure S used in our paper is based on the assessment of non-linear interactions of autonomic control loops of blood circulation and is promising for developing sensitive algorithms for the automatic classification of the REM sleep stage and the awake state. However, our analysis fails to discriminate between NREM and awake states, although such task is also important for the clinical and research practice. The indices *BRS* and *S* compared in our study showed different properties in distinguishing between the awake state and different stages of sleep. This is due to the different nature of these measures. Baroreflex sensitivity characterizes the changes in the response of baroreceptors caused by the changes in blood pressure. The result can be manifested in a change in the intensity of oscillations of the BP signal and RR-intervals in the LF-band. The index *S* characterizes a fundamentally non-linear effect of the phase coupling of oscillations in the loops of autonomic control of blood circulation. When calculating *S*, information on the dynamics of amplitudes of oscillations is not taken into account. For example, phase synchronization (and, accordingly, the index *S*) can increase with a decrease in the *LF*_*RR*_ oscillation power (and, accordingly, *BRS*), which can be seen from Table 1. Thus, the index *S* is not as a pure index of baroreflex sensitivity, but it provides important additional information on a non-linear interaction of the autonomic control loops. Therefore, in the studies of the dynamics of the autonomic control of blood circulation, the *S* and *BRS* indices do not replace, but complement each other, characterizing the features of the phase and amplitude dynamics, respectively, of analyzed systems. In the future, it looks promising to combine the advantages of the index *S* for detecting REM sleep with the capabilities of known approaches for classifying stages of NREM sleep, for example, using machine learning methods.

In this study, we analyze BP signals recorded by arterial catheterization in apnea patients. It is known that this pathology affects the processes of autonomic control (Guilleminault et al., 2005). Unfortunately, we are not able to compare the results of the analysis of patients and healthy subjects, since we do not have invasive BP records of healthy subjects. We expect that healthy subjects may have quantitative differences in *S* values compared to patients with apnea, but we assume that the conclusion about the differences in *S* values in different sleep stages and in awake state will remain valid. This assumption is based on the fact that we excluded from the analysis the signal epochs with apnea attacks. To clarify this important issue, we plan to compare the synchronization between the LF-components of RR-intervals and photoplethysmogram signals in apnea patients and in healthy subjects.

## Conclusion

The first methods for the assessment of human baroreflex function are based on statistical and spectral analysis of HR. However, such simple linear methods often lack the sensitivity to identify important features of this function in different physiological states. In these states, various systems of the body often change their properties simultaneously, demonstrating coherent and synchronous behavior. When studying such complex collective dynamics, non-linear methods of analysis have certain advantages. An example of such a non-linear method is the method based on the assessment of the degree of synchronization between the processes of autonomic control of HR rate and BP. This method exploits such a sensitive characteristic of the signal as its instantaneous phase. This approach allows us to identify the changes in the degree of coordinated behavior of the studied processes upon modulation of the baroreflex function by the processes of higher nervous activity.

We have found that in NREM sleep, the degree of synchronization of the LF-components of RR-intervals and BP signals is higher than in the awake state and lower than in REM sleep. The measure *S* may be promising for the development of algorithms for the automatic classification of sleep stages, especially for detecting REM sleep. The results of our study confirm the influence of the processes of higher nervous activity on the baroreflex function. Such influence can be considered in terms of modulation of the effective strength of coupling between the investigated loops of autonomic control.

## Data Availability Statement

The data analyzed in this study are subject to the following licenses/restrictions: These are data that belong to medical faculties and are not publicly available. Requests to access these datasets should be directed to TP, thomas.penzel@charite.de. The software that implements the method for diagnostics of synchronization developed by the authors is available at http://nonlinmod.sgu.ru/comprog_en.htm. It can be freely used in scientific research with reference to this work.

## Ethics Statement

The studies involving human participants were reviewed and approved by the Ethics Committee of Klinikum der Philipps-Universität Marburg, Germany. The patients/participants provided their written informed consent to participate in this study.

## Author Contributions

All authors contributed to writing and discussion of the manuscript.

## Conflict of Interest

The authors declare that the research was conducted in the absence of any commercial or financial relationships that could be construed as a potential conflict of interest.

## Publisher’s Note

All claims expressed in this article are solely those of the authors and do not necessarily represent those of their affiliated organizations, or those of the publisher, the editors and the reviewers. Any product that may be evaluated in this article, or claim that may be made by its manufacturer, is not guaranteed or endorsed by the publisher.

## Footnotes

1
http://nonlinmod.sgu.ru/comprog_en.htm
